# Immunopathological Analysis of a Mouse Model of Arthritis-Associated Scleritis and Implications for Molecular Targeted Therapy for Severe Scleritis

**DOI:** 10.3390/ijms23010341

**Published:** 2021-12-29

**Authors:** Yusuke Nishio, Hiroko Taniguchi, Ayaka Takeda, Junko Hori

**Affiliations:** Department of Ophthalmology, Nippon Medical School, Tama-Nagayama Hospital, Tokyo 2068512, Japan; y-nishio@nms.ac.jp (Y.N.); taniguchi@nms.ac.jp (H.T.); ayaka.takeda@gmail.com (A.T.)

**Keywords:** scleritis, animal model, collagen-II induced arthritis, posterior scleritis, molecular targeted therapy, biologic agent, TNF inhibitors, IL-6 inhibitors, anti-CD20, CTLA4Ig

## Abstract

Scleritis involves inflammation of the sclera, which constitutes 75% of the wall of the eye. This pathology is often seen as an ocular lesion associated with systemic inflammatory diseases. Severe types of scleritis such as posterior scleritis require urgent immunosuppressive treatments, including molecularly targeted therapies to avoid permanent visual impairment. Which molecules should be selected as targets has remained unclear. To clarify the pathogenesis of scleritis and propose appropriate target molecules for therapy, we have established novel animal model of scleritis by modifying the Collagen-II Induced Arthritis (CIA) model. Immunization twice with collagen II emulsified with complete Freund’s adjuvant (CFA) caused arthritis and scleritis. The clinical appearance resembled human diffuse scleritis. Histopathological analysis suggested that macrophages, plasma cells, deposition of immune complexes, and growth of blood and lymphatic vessels are involved in the pathogenesis of CIA-associated scleritis. In addition, we analysed the background diseases of posterior scleritis and responses to molecularly targeted therapies as a case series study. We inferred from both the animal model and case series study that targets should not be T cells, but factors inhibiting macrophage activity such as tumor necrosis factor (TNF) and interleukin (IL)-6, and molecules suppressing antibody-producing cells such as CD20 on B cells should be targeted by molecularly targeted therapies.

## 1. Introduction

The sclera constitutes 75% of the wall of the eye. The anterior outer surface of the sclera is covered by the conjunctiva through sparse connective tissue, and the anterior sclera is continuous with the corneal limbus. The scleral parenchyma mostly comprises extracellular matrix with components such as collagen fibres and elastic fibres, and the superior sclera is rich in blood vessels at the anterior part of the eye where it joins the Tenon capsule, subconjunctival tissue and external ocular muscles [1].

Scleritis involves inflammation of the sclera, including the episcleral vascular plexus lining the superficial layer of the sclera and the deep intrascleral vascular plexus, causing oedema and cellular infiltration of the sclera [2]. Non-infectious scleritis is the most common type [3], and is often seen as an ocular lesion associated with systemic inflammatory diseases [4]. In addition to severe ocular pain and redness, symptoms include radiating pain to the face, decreased visual acuity, and eye movement disorders. The Watson classification is commonly used to classify clinical findings of non-infectious scleritis, divided into episcleritis, anterior scleritis, and posterior scleritis [5]. Each of these is further subclassified as diffuse, nodular, or necrotising by shape, although episcleritis lacks the necrotising type [6]. 

Posterior scleritis is a rare but serious type of scleritis that can cause choroiditis and exudative retinal detachment, resulting in visual impairment, as well as ocular protrusion and eye movement disorders due to the spread of inflammation to the external ocular muscles [7]. The inflammatory changes in this pathology resemble those in anterior scleritis, but the scleritis may occur simultaneously from anterior to posterior or may occur at different times. Bilateral scleritis occurs in about 35% of cases [8], and systemic disease is not uncommon. B-mode ultrasonography shows scleral thickening, scleral nodules, dissection of the Tenon capsule from the sclera, and exophthalmos [9]. Optical coherence tomography (OCT) shows optic papillary oedema, choroidal folds at the posterior pole, and exudative retinal detachment, and imaging is useful for diagnosis and the evaluation of disease activity [10,11,12]. Anterior scleritis and uveitis may also be present [13,14,15].

In Japan, the frequency of scleritis associated with systemic autoimmune diseases is about 30% [10,16], and in Europe and the United States, it is reported to be 30–50% [17,18]. Wirnringa et al. reported that 32.7% of scleritis patients had a background disease, of which 29.8% were autoimmune diseases, most commonly rheumatoid arthritis, and 2.9% were infections due to herpes or syphilis. It was also reported that about 5% of patients with rheumatoid arthritis had episcleritis and about 2% had scleritis [2,4,19]. At the author’s institution, the frequency of scleritis accompanied by systemic autoimmune diseases was 22%, and the accompanying diseases were rheumatoid arthritis in 38.5%, followed by relapsing polychondritis and sarcoidosis in about 15% each. The percentage of new patients with scleritis among all first-time patients at our hospital was 0.3%, and the percentage among first-time patients with ocular inflammatory diseases was 13.6% [20].

Systemic inflammatory diseases associated with posterior scleritis include rheumatoid arthritis, anti-neutrophil cytoplasmic antibody (ANCA)-related vasculitis, and thyroiditis [21,22,23]. Posterior scleritis with visual loss requires urgent treatment to avoid permanent visual impairment. The choice of treatment depends on the severity of the ocular inflammation and associated systemic inflammatory diseases. Options include systemic steroids, immunosuppressive agents, and biologics, either alone or in combination [24,25,26,27]. As for molecularly targeted therapy with biologics for posterior scleritis, which molecules should be selected as targets remains unclear.

The pathogenesis of non-infectious scleritis has traditionally been inferred only from pathological investigations of biopsy samples and excised eyes [28,29,30,31], and animal models have been lacking. Since RA is the most common associated disease of scleritis and collagen is a component of sclera, we hypothesized that we could induce scleritis by modifying the CIA model, which is an animal model of RA. In this study, we infer factors that should be targeted by molecularly targeted therapy from case series study of posterior scleritis at our institution. In addition, we have established novel animal model of scleritis and performed a histopathological analysis of the pathogenesis.

## 2. Results

### 2.1. Mouse Model of Collagen II (CII)-Induced Arthritis-Associated Scleritis (CIA-Scleritis)

#### 2.1.1. Clinical Appearance and Histological Findings of CIA-Scleritis

**Figure 1:**
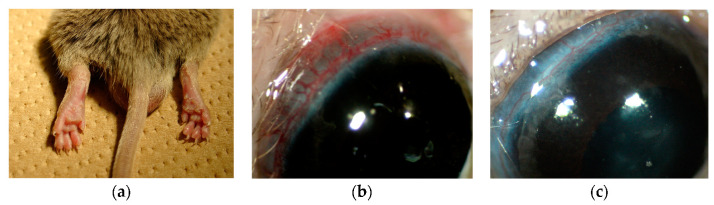
Clinical appearance of arthritis and scleritis in CIA-scleritis model. DBA/1J mice were immunized intradermally at the back neck with bovine 200µg of CII emulsified with CFA. On day 21, the mice were boosted by intradermal injection with 200µg of bovine CII emulsified with CFA around the eye. Clinical appearance of arthritis (**a**) and scleritis (**b**) at 3weeks of the 2nd immunization is shown. Normal eye of DBA/1J mice is shown as control (**c**).

In the standard CIA model using DBA/1 (*n* = 18), all the animals developed arthritis, but not scleritis. When the adjuvant of the second CII immunization was changed to CFA and injected around the eyes (*n* = 20), all the animals developed severe arthritis, followed by scleritis in all cases. Clinical findings comprised severe arthritis and dilation of scleral blood vessels (Figure 1a,b). Vessels of the sclera were definitively dilated compared with normal DBA/1J. (Figure 1b,c). The clinical appearance of CIA-scleritis resembled that of human diffuse scleritis.

**Figure 2:**
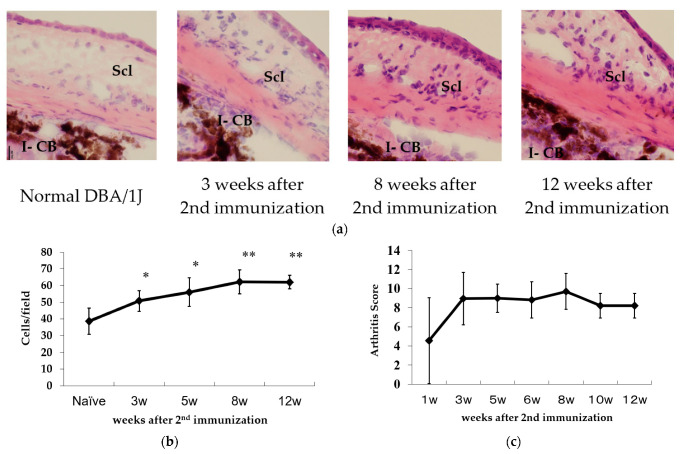
Infiltration of inflammatory cells into the sclera in CIA-scleritis model. The eyes of CIA-scleritis model were removed at 3–12 weeks after 2nd immunization. Cryostat sections of the eyes were stained with hematoxylin and eosin. Normal DBA/1J was used as control. “Scl” and “I-CB” denote sclera and Iris ciliary body, respectively. Original magnification, ×40 (**a**). The number of Infiltrating cells in scleral sections were counted (**b**). Data are the mean ± standard deviation of 3–6 eyes in each time point and were statistically compared with control (naïve) using the two-tailed Student’s *t* test (* *p* < 0.05, ** *p* < 0.001) (**b**). Arthritis was evaluated by Arthritis score for CIA model as below: 0 = Normal, 1 = Swelling of one digit, 2 = Swelling of two digits or more or swelling of the ankle or wrist, 3 = sever swelling of the entire paw (**c**).

Histological examination of the CIA-scleritis revealed that, in contrast with the normal DBA/1J mice, inflammatory cells were infiltrating into the anterior sclera, which was much thicker by 3 weeks after the second immunisation. At 8 weeks, the inflammatory process grew more severe, and inflammation remained for 12 weeks (Figure 2a). At 3 weeks after the second immunisation, the number of infiltrating cells in the anterior sclera of CIA-scleritis was significantly higher than that in normal DBA/1J mice (Figure 2b). In the CIA-scleritis model, severity of arthritis peaked at 3 weeks after the second immunisation. Inflammation of the sclera peaked later than arthritis (Figure 2b,c). We have not analyzed whether other organs were inflamed in these animals.

#### 2.1.2. Immune Cells, Complement, Immunoglobulin, and Hem- and Lymph-Angiogenesis in CIA-Scleritis

**Figure 3:**
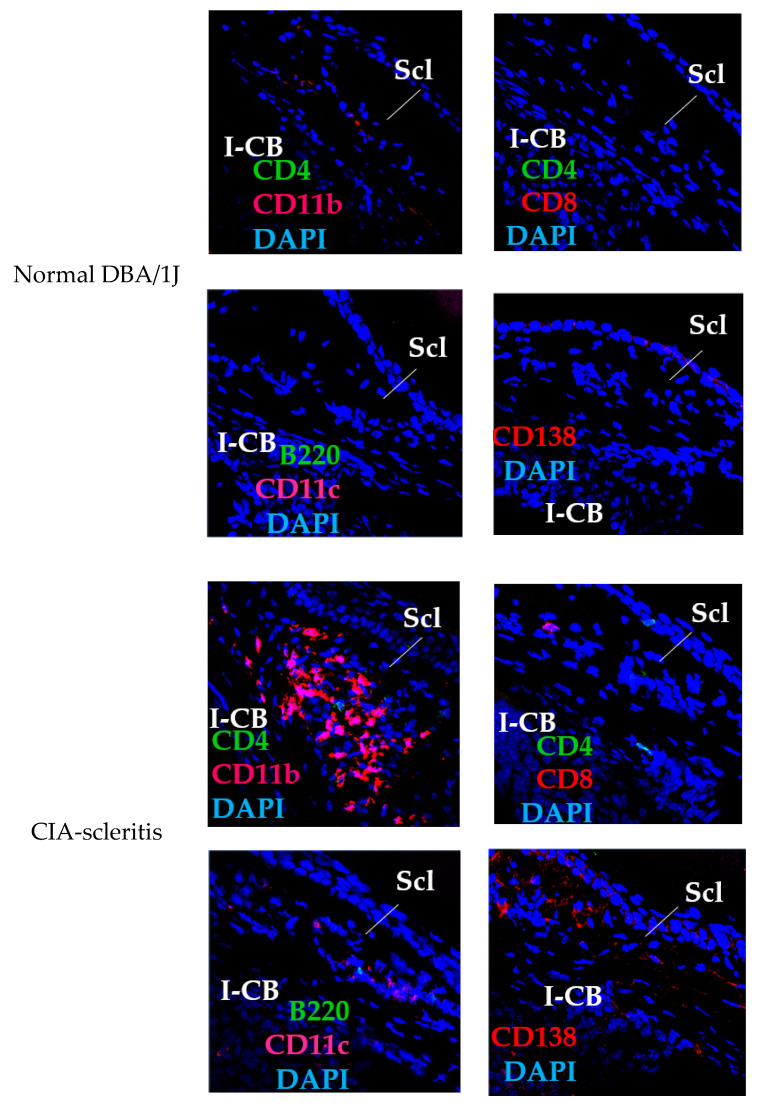
Infiltration of CD11b^+^, CD4^+^, CD8^+^, CD11c^+^, B220^+^, and CD138^+^ cells into the sclera in CIA-scleritis model. The eyes of CIA-scleritis model were removed at 3–8 weeks after 2nd immunization. Cryostat sections of the eyes were stained with FITC- or PE-conjugated anti-CD4, CD8, CD11b, CD11c, B220, or CD138 mAb. Nuclei were stained with DAPI. Normal DBA/1J was used as control. “Scl” and “I-CB” denote sclera and iris ciliary body, respectively. Original magnification, ×40.

**Figure 4:**
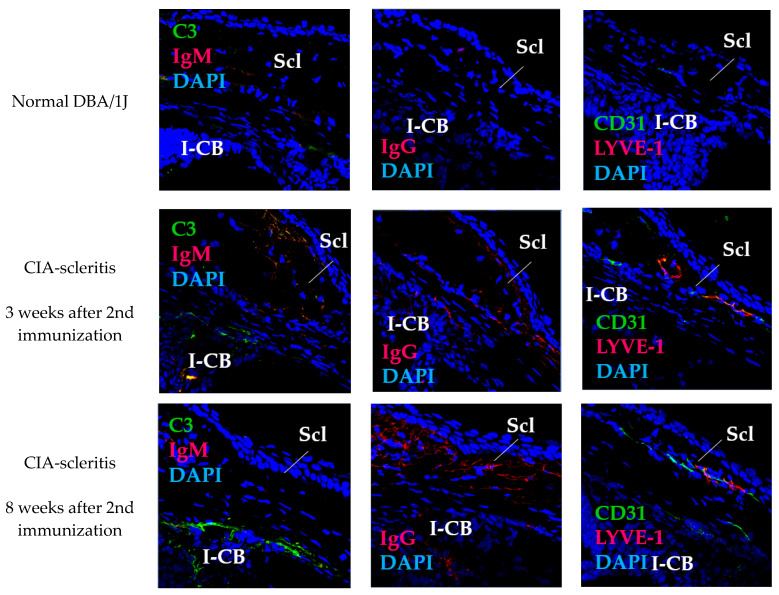
Expression of C3, IgM, IgG, CD31 and LYVE-1 in the sclera in CIA-scleritis model. The eyes of CIA-scleritis model were removed at 3–8 weeks after 2nd immunization. Cryostat sections of the eyes were stained with FITC-, PE-, or biotin-conjugated anti-C3, IgM, IgG, CD31, or LYVE-1 Ab. This was followed by staining with streptavidin-APC. Nuclei were stained with DAPI. Normal DBA/1J was used as control. “Scl” and “I-CB” denote sclera and iris ciliary body, respectively. Original magnification, ×40.

In sclera of the CIA-scleritis model, infiltration of CD4^+^, CD8^+^, CD11b^+^, CD11c^+^, B220^+^ and CD138^+^ cells was observed from 3 weeks after the second immunisation with CII (Figure 3). Among these immune cells, CD11b^+^ and CD138^+^ cells infiltrated more, and CD4^+^, CD8^+^, and CD11c^+^ cells infiltrated less. Deposition of complement (C3), immunoglobulin (IgG and IgM), and blood and lymphatic growth markers (CD31/PECAM1, panendothelial marker; and LYVE-1, lymphatic endothelial marker) was particularly evident in the region of anterior sclera in contact with the ciliary body (Figure 4). Thus, macrophages, plasma cells (antibody-producing cells), immunocomplex deposition, and blood and lymphatic vessel growth are suggested to be involved in the pathogenesis of CIA-scleritis.

### 2.2. Case Series Study of Posterior Scletritis

The subjects included one man and six women, with a mean age of 66.1 years (range, 40–88 years). Four cases were unilateral and three cases were bilateral (Table 1). Ocular pain was present in all patients. High intraocular pressure was observed in three patients (Table 2). Mean logMAR of visual acuity before treatment was 0.4 (range, −0.1 to 1.5), equivalent to 0.4 (range, 1.2–0.03) in decimal visual acuity. Mean post-treatment logMAR of visual acuity was 0.2 (range, −0.2 to 1.8), equivalent to 0.6 (20-cm hand motion −1.5) decimal visual acuity (Table 3).

Five patients showed anterior scleritis, and 2 patients had anterior chamber inflammation. Retinal vein anastomosis was not seen in any of the patients. Papillary swelling was seen in one case. Exudative retinal detachment was observed in all patients (Table 2). Systemic steroids were administered in all cases, with pulse therapy used in four cases. Two cases achieved remission with 30–40 mg oral steroids alone. Four patients were treated with oral immunosuppressive drugs, with two patients subsequently recovering. We present three cases in whom biologics were used for molecularly targeted therapy (Table 3).

#### 2.2.1. Case Presentations of Severe Posterior Scleritis Treated with Molecularly Targeted Therapies

##### Case 1

A 40-year-old woman with a complaint of right oculomotor pain and decreased visual acuity was referred to our department by her primary care physician in April X. On the first visit to our department, her best corrected visual acuity (BCVA) was 20/25 in the right eye, and no evidence of intraocular inflammation was seen.

**Figure 5:**
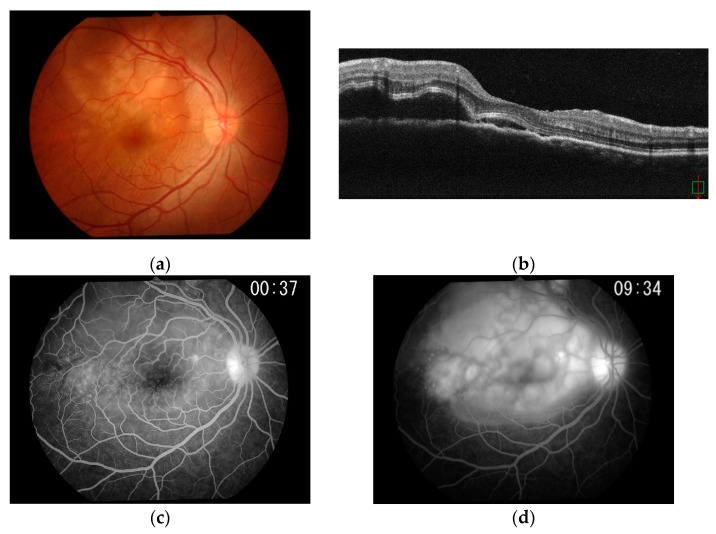
Case 1: Colour fundus photograph, OCT, and FFA of the right eye. (**a**) Colour fundus montage photograph of the right eye shows subretinal fluid and multiple scattered, discrete areas of choroiditis. (**b**) OCT of the right eye shows extensive SRD and a chorioretinal fold. (**c**) FFA of the right eye in the early phase shows subretinal granular hyperfluorescence. (**d**) FFA of the right eye in the late phase shows strong subretinal leakage and accumulation with extensive SRD.

Fundus examination of the right eye showed subretinal fluid and multiple scattered, discrete areas of choroiditis (Figure 5a). OCT showed extensive serous retinal detachment (SRD) and chorioretinal fold in the right eye (Figure 5b). The later stages of fundus fluorescein angiography (FFA) revealed strong subretinal leakage and accumulation with extensive SRD in the right eye, as well as early subretinal granular hyperfluorescence. Posterior scleritis was therefore diagnosed (Figure 5c,d).

**Figure 6:**
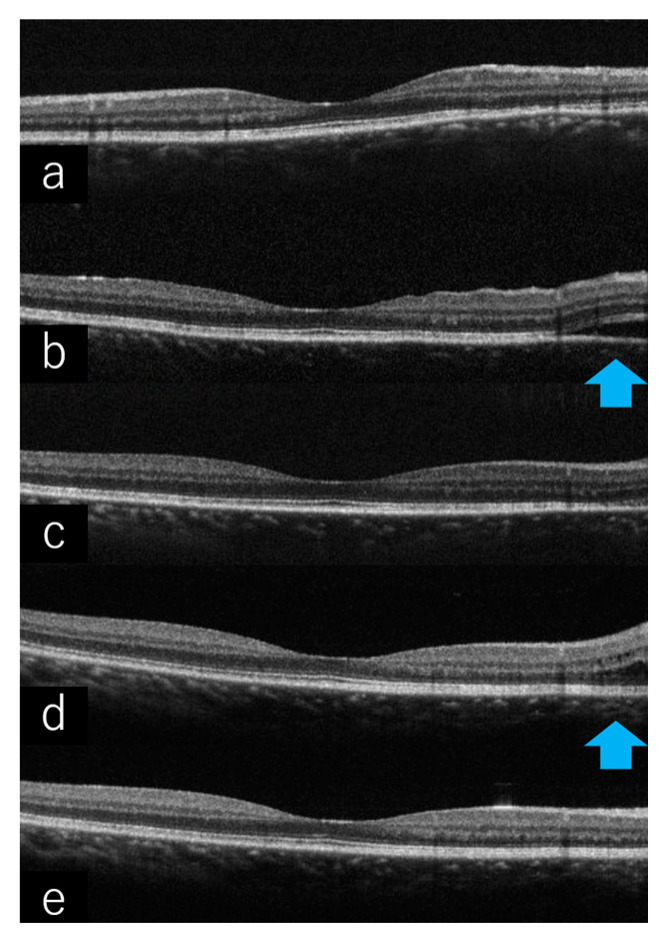
Case 1: OCT of the right eye. (**a**) Posterior scleritis quickly disappeared after starting pulsed steroid therapy. (**b**) Posterior scleritis relapsed after tapering to 5 mg/day of PSL. (**c**) The dose of PSL was increased to 20 mg/day and 100 mg/day of CysA was added with once remission was achieved. (**d**) While taking 10 mg/day of PSL and 175 mg/day of CysA, scleritis relapsed. (**e**) After introducing ADA, the patient has remained in remission.

To rule out rheumatoid arthritis, ANCA-associated vasculitis, relapsing polychondritis, thyroid disease, sarcoidosis, toxoplasmosis, and tuberculosis, complete blood count, immunoglobulins (IgA, IgG, IgM), protein fractions, C-reactive protein (CRP), angiotensin converting enzyme (ACE), antinuclear antibodies, complement titer, rheumatoid factor, thyroxine (T4), triiodothyronine (T3), thyroid stimulating hormone (TSH), anti-thyroglobulin antibodies, anti-thyroid peroxidase (TPO) antibodies, anti-TSH receptor antibodies, toxoplasma antibodies, ANCA, chest X-ray, and tuberculin reaction tests were performed. With a systemic examination revealing no background of systemic immune disease, oral prednisolone (PSL) was started at 60 mg/day after she had been treated with pulsed steroid therapy with 1 g/day of PSL for 3 consecutive days from April X. Posterior scleritis quickly resolved (Figure 6a). In August X, after tapering to 5 mg/day of PSL, posterior scleritis relapsed (Figure 6b). The dose of PSL was increased to 20 mg/day (0.4 mg/kg/day) and 100 mg/day (2 mg/kg/day) of cyclosporin (CysA) was added with once remission was achieved (Figure 6c). While the patient was taking 10 mg/day of PSL and 175 mg/day of CysA in April X+1, scleritis relapsed (Figure 6d). We tried to increase the dose of CysA, but the trough level was not elevated at about 60–70 ng/mL with no underlying disease in the screening test, so adalimumab (ADA) was introduced in our department in June X+1 in collaboration with the Department of Rheumatology. After introducing ADA, ocular inflammatory findings improved markedly and she has since remained in remission (Figure 6e).

##### Case 2

An 81-year-old woman presented with a complaint of redness and pain in the right eye. She had been diagnosed with rheumatoid arthritis (RA) at 67 years old and was being treated with PSL and methotrexate (MTX) by her local physician. Abatacept (CTLA4Ig) had been introduced when the RA was poorly controlled, but was discontinued when pneumocystis pneumonia developed during the course of treatment. At this point, she was referred to the Department of Rheumatology. She was referred to our department in March Y after noticing redness and pain in the right eye when taking 4 mg/day of PSL and 2 mg/day of tacrolimus.

**Figure 7:**
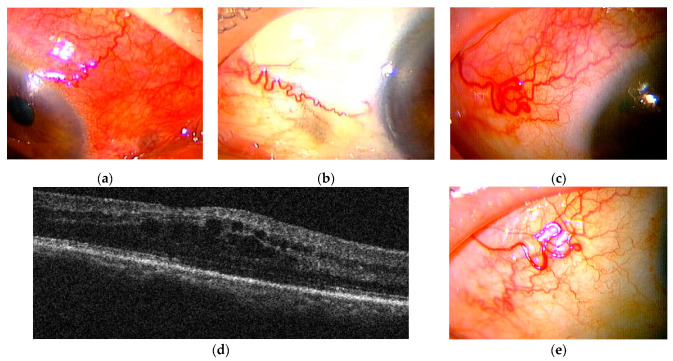
Case 2: Clinical appearance of anterior diffuse scleritis and OCT in the right eye. (**a**) Clinical appearance of anterior diffuse scleritis in the right eye at the first visit. (**b**) Scleritis persisted for about 6 months even after topical treatment of eye drops and SCTA. (**c**,**d**) One week after initiating CTLA4Ig, scleritis in the right eye worsened and macular oedema developed with an alleged paradoxical reaction. (**e**) After introducing GLM, scleritis rapidly disappeared within 1 month.

Diffuse scleritis of the right eye associated with RA was diagnosed (Figure 7a). Four eye drops of betamethasone 0.1%, five eye drops of immunosuppressant, and 200 mg of celecoxib each day did not relieve the inflammation, and scleritis persisted for about 6 months even after subconjunctival injection of 0.1 mL of triamcinolone acetonide (SCTA) (Figure 7b).

**Figure 8:**
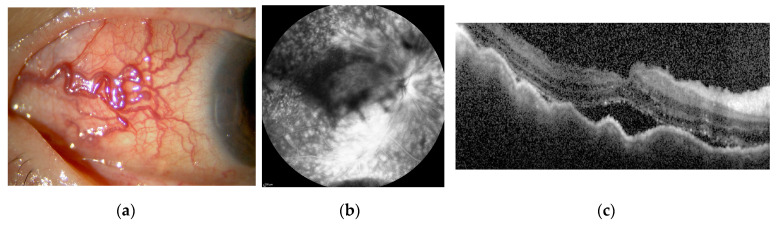

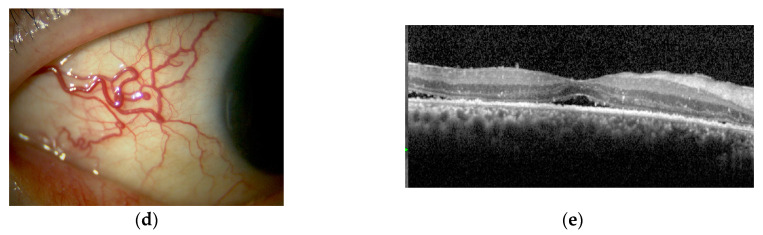
Case 2: Clinical appearance of anterior diffuse scleritis, FFA and OCT in the right eye (**a**–**c**) Scleritis relapsed, and the dose of GLM was doubled, but diffuse scleritis in the right eye did not go into remission. The patient also developed posterior scleritis in the right eye. (**d**,**e**) After bio-switching to SAR, scleritis rapidly disappeared. The patient remains in remission.

The rheumatology department was consulted regarding treatment of this patient with biologics. Because of the history of pneumonia, she was started on CTLA4Ig, a drug with a lower risk of serious infections than TNF inhibitors, in November Y. However, one week after introducing CTLA4Ig, scleritis in the right eye worsened and macular oedema became apparent. This was considered a paradoxical reaction (Figure 7c,d). Ten weeks after introducing CTLA4Ig, the patient was switched to golimumab (GLM), a TNF inhibitor, by the department, and scleritis rapidly disappeared within one month (Figure 7e). The scleritis relapsed in December Y+1, and the dose of GLM was doubled in January Y+2, but diffuse scleritis in the right eye did not go into remission. In March Y+2, the patient also developed posterior scleritis in the right eye (Figure 8a–c). Sub-Tenon injection of 0.5 mL of triamcinolone acetonide (STTA) into the right eye proved unsuccessful. She consulted the rheumatology department for consideration of 30 mg/day PSL, but was bio-switched to the IL-6 inhibitor sarilumab (SAR) in June Y+2 due to the high risk of infection and a history of synovitis and psoriasis induced by TNF inhibitors. Scleritis rapidly resolved and the patient has since remained in remission (Figure 8d,e).

##### Case 3

An 85-year-old woman with a complaint of hyperaemia on the nasal side of the left eye presented to her primary care physician in October Z-2, and was followed-up with steroid drops. As her condition remained unimproved, she was referred to our department in April Z-1. Nodular scleritis was diagnosed and improved with frequent eye drops of betamethasone 0.1%, five eye drops of immunosuppressant, 200 mg of celecoxib, and 0.1 mL of SCTA, resulting in a reverse referral in July Z-1. After she moved to another location, inflammation relapsed, so she returned to her previous physician and was referred back to us on March 2nd, Z-1.

**Figure 9:**
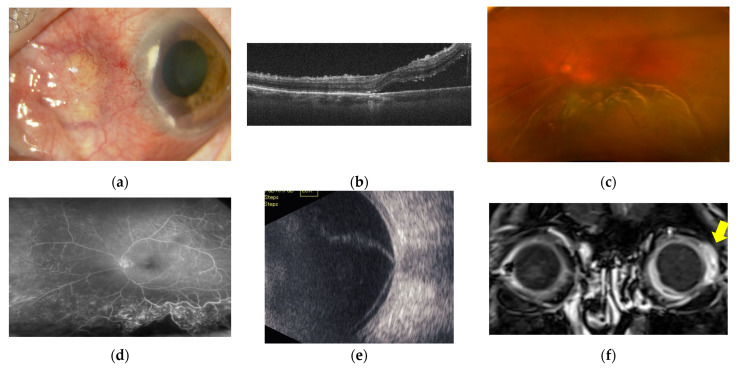
Case 3: Clinical appearance of anterior necrotizing scleritis, OCT, Colour fundus imaging, FFA, B-mode ultrasonography and MRI in the right eye. (**a**) Photograph of the anterior segment of the left eye at the first visit, showing necrotising scleritis and corneal marginal infiltration. (**b**) OCT shows the neuroepithelial layer as raised and wrinkled, with underlying fluid. (**c**) Colour fundus imaging shows papilloedema and extensive SRD including the fovea. (**d**) FFA shows retinal vasculitis consistent with SRD. (**e**) B-mode ultrasonography reveals increased scleral thickening (T-signatures) and extensive SRD. (**f**) Contrast-enhanced MRI shows contrast enhancement localised to the ocular wall of the left eye (yellow arrows).

BCVA of the left eye was 20/667 at the time of the first visit, and the left eye showed intense panuveitis with anterior chamber (AC) cell grade 3+ and opacitas corporis vitrei (OCV) grade 1+ according to national eye institute/SUN working group (NEI/SUN) classification. A photograph of the anterior segment of the left eye showed necrotising scleritis and corneal marginal infiltration (Figure 9a). The left eye displayed papilloedema and extensive SRD including the fovea (Figure 9c), and OCT showed extensive SRD and dilated choroidal vessels (Figure 9b). FFA showed retinal vasculitis consistent with SRD (Figure 9d), B-mode ultrasonography showed T-signatures and extensive SRD (Figure 9e), and contrast-enhanced magnetic resonance imaging (MRI) showed contrast enhancement of the ocular wall (Figure 9f).

**Figure 10:**
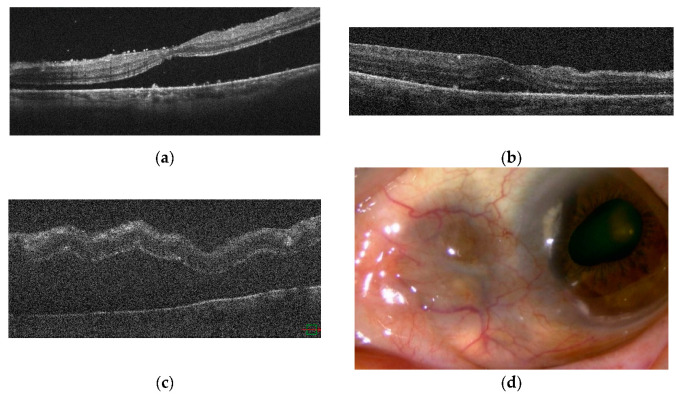
Case 3: OCT and clinical appearance of anterior necrotizing scleritis in the right eye. (**a**) OCT after introduction of 30 mg of PSL, showing worsened SRD. (**b**) After four courses of steroid pulse therapy and RTX, scleritis temporarily resolved. (**c**) OCT at the time of reducing the PSL dose to 12.5 mg/day shows relapse of scleritis. (**d**) Anterior segment of the left eye after 250 mg/day PSL pulse therapy and SCTA, showing continued scleral thinning and melting.

Screening blood samples were positive for PR3-ANCA, and necrotising anterior scleritis and posterior scleritis associated with granulomatosis with polyangiitis were diagnosed. The patient was started on 30 mg/day (0.6 mg/kg/day) of PSL on March 2nd, Z. However, SRD worsened and inflammation was poorly controlled (Figure 10a), so steroid pulse therapy (1 g/day of PSL for 3 consecutive days) was started on 25 March, and oral PSL was started at 40 mg/day. The patient was referred to the department of rheumatology for consideration of immunosuppression, including cyclophosphamide, but the risk of infection considered too high, so the department instead chose to introduce rituximab (RTX), a single cytokine inhibitor, for four courses starting on March 28. After four courses of steroid pulse therapy and RTX, scleritis temporarily resolved and visual acuity was improved (Figure 10b).

However, in May Z, when PSL was tapered to 12.5 mg/day, scleritis relapsed and visual acuity in the left eye decreased to 40 cm (m.m) (Figure 10c). At the same time, her general condition deteriorated, including low IgG and cytomegalovirus (CMV) positivity with pronounced side effects of immunosuppressive therapy, resulting in administration of 250 mg of PSL and high-dose immunoglobulin therapy in June Z. As steroid pulse therapy proved unsuccessful and the side effects of RTX prevented additional immunosuppressive treatment, SCTA was performed on the necrotic area and contralateral side in June Z despite the limited effect on posterior scleritis. SCTA did not show any effect, and scleral thinning and melting continued (Figure 10d). The department of rheumatology is currently planning to apply TNF inhibitors and other drugs while waiting for an improvement in her immune status.

## 3. Discussion

In this study, we have described the development of novel animal model of scleritis. Conventional pathological analysis of scleritis has relied on histopathological analysis of clinically removed eyes. The histopathological findings from these clinically removed eyes represent the terminal results of unhealed scleritis modified by various treatments [32,33,34,35]. Such methods of analysis are inadequate to achieve a sound understanding of the pathogenesis underlying scleritis. Although various reports have described the use of molecularly targeted therapies using biologics such as TNF inhibitors, IL-6 inhibitors, CTLA4Ig, anti-CD20 antibody, and anti-CD25 antibody, the optimal molecules to target with molecularly targeted therapy in scleritis have remained unknown [36,37,38,39,40]. Against this background, immunopathological analysis using animal models of scleritis is necessary.

CIA is currently the most used animal model of RA involving autoimmune responses to specific antigens. Since CIA are MHC constrained (I-Aq, DBA/1 mice), DBA/1 mice are used in the standard protocol. CIA do not have many extra-articular symptoms. We succeeded in inducing scleritis by modifying CIA. In this model, arthritis is followed by the development of scleritis. The immune cells infiltrating the sclera were predominantly CD11b^+^, with some (but not much) CD4^+^ T and CD8^+^ T cell infiltration. B cells, and plasma cells in particular, were predominantly seen infiltrating, with complement and immunoglobulin deposition, as well as hem- and lymph-angiogenesis. In other words, the innate immune response of macrophages, antibody production from plasma cells, and deposition of complement and immune complexes induces vascular lesions in the early stages of scleritis. From these findings, we infer that TNF, which acts to suppress macrophages, and B-cell suppression offer more promising targets for therapy than T-cell suppression. In addition to elucidating the pathogenesis, it will be possible to administer various molecularly targeted drugs to this model and evaluate their effects.

Vasculitis of scleral blood vessels has also been reported in pathological images of excised eyes with non-infectious necrotizing scleritis, and various immune cell infiltrations in the region, ischemic scleral necrosis due to fibrinoid necrosis and vascular occlusion of the vessel wall, collagen destruction by inflammatory cells, and collagenolysis due to unbalanced expression of matrix metalloproteinases (MMPs) and tissue inhibitors of metalloproteinases (TIMPs) have been reported [29,33,41,42]. These findings are the end result of necrotizing scleritis. In contrast, our CIA-scleritis model shows the pathology in the early stages of the disease, with macrophages and antibody-producing cells predominating in the infiltrate, presence of complement and immune complexes, and angiolymphogenesis as newly discovered early pathological findings.

It has been reported that the local area of arthritis in CIA is infiltrated with macrophages, T cells and B cells [43,44,45]. Pathology studies of arthritis in patients with rheumatoid arthritis also showed infiltration of T and B cells, macrophages, and neutrophils [46], suggesting that the pathology of both types of arthritis is similar. Arthritis in CIA models has been reported to be ameliorated by the administration of immunosuppressive drugs and antibodies to cytokines. In particular, inhibition of IL1, IL-6, and TNF have been reported to be highly effective in suppressing arthritis. These cytokines target macrophages, and from the results of analysis of the CIA scleritis model in this study, it is possible that inhibition of IL1, IL-6, and TNF might also be effective in suppressing scleritis. 

In the case series study presented here, we analysed the background pathologies in cases of posterior scleritis, as the most severe form of scleritis, and responses to molecularly targeted therapies. Among three cases inadequately treated with steroids, one case was free of systemic inflammatory disease and responded well to TNF inhibitors. The other two cases were associated with RA and ANCA-associated vasculitis, respectively, and were selected by the department of rheumatology to receive molecularly targeted agents other than TNF inhibitors, because of their advanced age and poor general condition.

Another report has described paradoxical reactions to CTLA4Ig resulting in exacerbation of scleritis, suggesting that CTLA4Ig, which suppresses only T cells, is inappropriate as a molecularly targeted therapy for scleritis [47]. In that case, the patient was switched from CTLA4Ig to a TNF inhibitor, which proved successful for a while, but became ineffective over the long term, and was switched to an IL-6 inhibitor, resulting in remission. The fact that marked infiltration of macrophages into the sclera was seen in our mouse model of scleritis suggests that TNF and IL-6, as cytokines produced by macrophages for innate immunity, represent appropriate target molecules. In fact, among the various reports of molecularly targeted therapies for scleritis, those describing the efficacy of TNF inhibitors are the most common [48,49,50,51,52,53].

In the case where an anti-CD20 antibody (rituximab) was selected, scleritis improved initially, but then recurred. Because the anti-CD20 antibody targets B cells, the patient became immunocompromised with prolonged hypoimmunoglobulinaemia after treatment with this drug, and was unable to resume the next molecularly targeted therapy for scleritis. In our mouse model of scleritis, the sclera showed infiltration of B cells and plasma cells, as well as IgG deposition, suggesting that targeting antibody-producing B cells is appropriate. On the other hand, compared to single cytokine inhibition, the side effects of severe immunodeficiency, such as hypoimmunoglobulinaemia, appear quite disadvantageous.

In summary, our mouse model of scleritis associated with autoimmune arthritis displayed infiltration of macrophages, B cells, and plasma cells into the sclera, deposition of antibodies and complement, and hem- and lymph-angiogenesis in the sclera. In severe cases of clinical scleritis, molecularly targeted therapies that suppress T cells not only proved ineffective, but also exacerbated scleritis, while inhibitors of TNF and IL-6, which are involved in macrophage function, and B-cell inhibition were effective and consistent with the pathology implied by the animal model. Our mouse model of scleritis may be useful not only for analysing the pathogenesis of the disease, but also for inferring the therapeutic effects of various molecularly targeted agents, and we are conducting further immunological analysis using this model. There have been no previous reports of the development of scleritis in mice that develop spontaneous autoimmunity as far as we have been able to find, but this might be because the eyes were not fully analyzed in each model. This is also an issue that should be verified in the future.

## 4. Materials and Methods

### 4.1. Mouse Model of CIA-Scleritis

#### Mice and Anesthesia

Male DBA/1J mice were purchased from Sankyo Lab Service (Tokyo, Japan) and used at 8–10 weeks old. Treatment was performed in accordance with the Association for Research in Vision and Ophthalmology guidelines on the use of animals in research. The protocol for this animal study was reviewed and approved by our institutional review committee. Before all surgical procedures, each mouse was anaesthetised by intramuscular injection of a mixture of 3.75 mg of ketamine and 0.75 mg of xylazine.

### 4.2. Abs

For fluorescence immunohistochemistry or flow cytometry, antibodies (Abs) against mouse CD3 (145-2C11, Armenian hamster IgG), CD4 (GK1.5, rat IgG2b), CD8 (53.6.72, rat IgG2a), CD11b (M1/70, rat IgG2b), B220 (RA3-6B2, rat IgG2a), CD11c (N418, Armenian hamster IgG), CD138 (281-2, rat IgG2a), C3 (5F9, rat IgG2a), IgG (Polyclonal, Goat IgG), IgM (Polyclonal, Goat IgG), CD31 (MEC13.3, rat IgG2a), and LYVE-1 (Polyclonal, rabbit IgG) were used. All fluorescein isothiocyanate (FITC)-, phycoerythrin (PE)-, allophycocyanin-, and biotin-conjugated mAbs and isotype control Igs were obtained from eBioscience (San Diego, CA, USA).

### 4.3. CIA-Scleritis

To establish a scleritis model, we modified the CIA model as a mouse model of autoimmune polyarthritis with similarities to human RA. 

Male DBA/1J mice at 8 weeks old were immunised intradermally at the back of the neck with 200 μg of bovine CII in 0.05-M acetic acid, emulsified with an equal volume of complete Freund’s adjuvant (CFA, containing 100 μg of H37Ra *Mycobacterium tuberculosis*). On day 21, mice received an intradermal booster injection with 200 μg of bovine CII emulsified with CFA around the eye. Arthritis and anterior scleritis were induced within 1–3 weeks after the second immunisation.

### 4.4. Evaluation of Arthritis

Arthritis was evaluated using the arthritis score for the CIA model, as follows: 0 = normal; 1 = swelling of one digit; 2 = swelling of two digits or more, or swelling of the ankle or wrist; and 3 = severe swelling of the entire paw [54].

### 4.5. Evaluation of Scleritis

Clinical appearance of the eyes in CIA-scleritis mice was observed under the surgical microscope. The number of infiltrating cells in scleral sections were counted using the original magnification (×40). The masked cell count assessment was performed by a single observer (H.T.). Eyes were harvested at 3, 5, 8 or 12 weeks after the second immunisation. Frozen sections were prepared for haematoxylin and eosin staining and immunofluorescence staining (CD4, CD8, B220, CD11b, CD11c, CD138, C3, IgG, IgM, CD31, and LYVE-1).

### 4.6. Histology and Immunohistochemistry

For immunohistochemistry, eyes were removed and frozen in OCT compound (Sakura Finetechnical, Tokyo, Japan) in acetone-dry ice and stored at −80 °C. Cryostat sections (5 μm; approximately 20 sections per eye) were fixed in cold acetone, followed by immunofluorescent staining for the detection of mouse CD4, CD8, B220, CD11b, CD11c, CD138, C3, IgG, IgM, CD31, LYVE-1, and rat IgG (eBioscience). Other sections were stained with haematoxylin and eosin. Briefly, after blocking with 2% bovine serum albumin, sections were incubated with PE-, FITC-, or biotin-conjugated primary Ab diluted to 4 µg/mL for 2 h. This was followed by staining with streptavidin-allophycocyanin (APC) (eBioscience) diluted to 4 µg/mL for 1 h at room temperature. After washing with phosphate buffered saline (PBS), sections were mounted with 4,6-diamidino-2-phenylindole-containing mounting medium and observed under confocal microscopy (LSM710; Zeiss, Jena, Germany).

### 4.7. Statistical Analyses

Infiltrating cells were analysed using the two-tailed Student’s *t* test. Probability (*p*) values < 0.05 were considered statistically significant.

### 4.8. Case Series Study

A retrospective study of medical records was conducted for seven patients with posterior scleritis who visited the Ocular Inflammation Outpatient Clinic of Nippon Medical School Tama Nagayama Hospital between 2018 and 2020. This study was approved by the Ethics Committee of Nippon Medical School Tama Nagayama Hospital in accordance with the Declaration of Helsinki. The age, sex, bilateral or unilateral scleritis, associated systemic diseases, ocular findings, treatments, recurrence, and visual outcomes of patients were analysed. Three cases that received molecularly targeted therapy using biologics were described in detail, including the course of treatment and response to each targeted drug.

## Figures and Tables

**Table 1:** Demographic characteristics of patients.

Case No.	Gender	Age at Ocular Inflammation Onset	Age of Posterior Scleritis Onset	Follow-Up (Month)	Laterality	Associated Systemic Disease
1	M	81	82	23	B	−
2	F	76	76	19	B	−
3	F	31	37	165	B	−
4	F	85	85	25	R	−
5	F	36	36	52	R	−
6	F	75	80	29	R	RA
**7**	F	82	83	28	L	AAV

**Table 2:** Symptoms and clinical findings.

Case No.	Ocular Pain	Associated Anterior Scleritis	Anterior Chamber Cells	Serous Retinal Detachment	RPE Folds	Optic disc Swelling/Hyperemia	Ocular Hypertension (>21 mmHg)
1	+	+	−	+	+	−/−	+
2	+	−	−	+	+	+/+	−
3	+	+	−	+	+	−/−	+
4	+	+	+	+	+	−/−	+
5	+	−	−	+	+	−/−	−
6	+	+	−	+	+	−/−	−
**7**	+	+	+	+	+	−/−	−

**Table 3:** Treatment, recurrences, and visual outcomes.

Case No.	Topical Treatment		Systemic Treatment					Recurrences (Number)	Per- sistent	BCVA before Treatment OD, OS	BCVA after Treatment OD, OS
	Eye Drops	Subconjunctival Injection of Steroid	Oral NSAIDs	Oral Steroid	Steroid Pulse Therapy	Immune Suppressant	Biologics				
1	+	−	+	+	+	−	−	0	−	20/500, 20/32	20/12, 20/12
2	+	−	+	+	+	+	−	2	−	20/20, 20/32	20/16, 20/20
3	+	+	+	+	−	+	−	5	−	20/32, 20/25	20/12, 20/20
4	+	+	+	+	−	−	−	0	−	20/32, 20/25	20/40, 20/20
5	+	−	+	+	+	+(CysA)	+(ADA)	2	−	20/25, 20/16	20/12, 20/16
6	+	+	+	+	−	+(MTX, TAC)	+(CTLA4Ig → GLM →SAR)	1	−	20/1000, 20/32	20/600, 20/20
7	+	+	+	+	+	−	+(RTX)	1	+	20/25, 20/600	20/25, 20 cm HM
Total	7/7 (100%)	4/7 (57%)	7/7 (100%)	7/7 (100%)	4/7 (57%)	4/7 (57%)	3/7 (43%)				

NSAIDs non-steroid anti-inflammatory drugs, BCVA best corrected visual acuity, + present, −absent, CysA cyclosporine, MTX methotrexate, TAC tacrolimus, ADA Adalimumab, CTLA4Ig abatacept, GLM golimumab, SAR Sarilumab, RTX rituximab.

## Abbreviations

TNF: Tumor Necrosis Factor, IL: interleukin, CIA: type II Collagen Induced Arthritis, CII: type II collagen, CFA: complete Freund’s adjuvant, OCT: Optical coherence tomography, BCVA: best corrected visual acuity, SRD: serous retinal detachment, ANCA: anti-neutrophil cytoplasmic antibody, Fig: figure, FFA: fundus fluorescein angiography, PSL: prednisolone, NSAIDS: non-steroid anti-inflammatory drugs, CysA: cyclosporine, MTX: methotrexate, TAC: tacrolimus, ADA: Adalimumab, CTLA4Ig: abatacept, GLM: golimumab, SAR: Sarilumab, RTX: rituximab, RA: rheumatoid arthritis, STTA: sub-Tenon’s injection of triamcinolone acetonide, SCTA: subconjunctival injection of triamcinolone acetonide, AC: anterior chamber, OCV: opacitas corporis vitrei, NEI/SUN: national eye institute/SUN working group, MRI: magnetic resonance imaging, CMV: cytomegalovirus, FITC: fluorescein isothiocyanate, PE: phycoerythrin, APC: allophycocyanin.
